# An empirical examination of the mislabelling of fat as an emotion in sub-clinical eating disorder groups

**DOI:** 10.1186/2050-2974-2-S1-O60

**Published:** 2014-11-24

**Authors:** Yichelle Zhang, Stephen Touyz, Bruce Burns, Lenny Vartanian, Maree Abbott

**Affiliations:** 1 The University of Sydney, Sydney, Australia; 2 The University of New South Wales, Sydney, Australia

## 

"I feel fat" is a statement women, and increasingly men, often make. Clinical observations indicate that these feelings of fat are experienced more intensely and frequently in those who suffer from eating disorders. However, physical fat is not an emotion that one can "feel". According to body displacement theory, the propensity to mislabel fat as an emotion is a displacement of other affect, but there has been little empirical evidence to test this theory. Consequently, the current study aims to investigate this phenomenon by examining the impact of manipulating both negative and positive affect on the propensity to feel fat. In addition, this study will examine whether bodily sensations may also play a role in driving the mislabelling of feeling fat. The results from this study will provide greater insight into the drivers of feelings of fat. In addition, this study will address a common assumption that feelings of fat in people with eating disorders (especially those who are emaciated) are considered to be irrational, when such feelings of fat may be a substitution of other emotions and/or bodily sensations. Together, these findings could potentially contribute to altering strategies in the treatment of eating disorders.

This abstract was presented in the **Disordered Eating and Body Image** stream of the 2014 ANZAED Conference.

